# A Phosphatidyl Conjugated Telomerase-Dependent Telomere-Targeting Nucleoside Demonstrates Colorectal Cancer Direct Killing and Immune Signaling

**DOI:** 10.3390/biom14121616

**Published:** 2024-12-18

**Authors:** Merve Yilmaz, Sibel Goksen, Ilgen Mender, Gunes Esendagli, Sefik Evren Erdener, Alessandra Ahmed, Ates Kutay Tenekeci, Larisa L. Birichevskaya, Sergei M. Gryaznov, Jerry W. Shay, Z. Gunnur Dikmen

**Affiliations:** 1 Department of Medical Biochemistry, Faculty of Medicine, Hacettepe University, Ankara 06100, Turkey; merve.yilmaz@utsouthwestern.edu (M.Y.); atesktenekeci@gmail.com (A.K.T.); gunnur@hacettepe.edu.tr (Z.G.D.); 2 Department of Cell Biology, UT Southwestern Medical Center, Dallas, TX 75390, USA; alessandra.ahmed@utsouthwestern.edu; 3 Department of Medical and Surgical Research, Institute of Health Sciences, Hacettepe University, Ankara 06100, Turkey; goksensibel@gmail.com (S.G.); gunese@hacettepe.edu.tr (G.E.); 4 MAIA Biotechnology, Inc., Chicago, IL 60606, USA; imender@maiabiotech.com (I.M.); sgryaznov@maiabiotech.com (S.M.G.); 5 Department of Basic Oncology, Cancer Institute, Hacettepe University, Ankara 06100, Turkey; 6 Neurological and Psychiatric Research and Application Center, Faculty of Medicine, Hacettepe University, Ankara 06100, Turkey; evrenerdener@hacettepe.edu.tr; 7 Institute of Microbiology National Academy of Sciences, 220084 Minsk, Belarus; larbir@mail.ru

**Keywords:** cancer, telomerase, telomere-targeted therapy, 6-thio-dG, phosphatidyl lipid conjugated 6-thio-dG

## Abstract

Telomerase and telomeres are crucial in cancer cell immortalization, making them key targets for anticancer therapies. Currently, 6-thio-dG (THIO) combined with the anti-PD-1 inhibitor Cemiplimab is under phase II clinical investigation (NCT05208944) in NSCLC patients resistant to prior immunotherapies. This study presents the design, synthesis, and evaluation of novel bimodular conjugate molecules combining telomere-targeting nucleoside analogs and phosphatidyl diglyceride groups. Among them, dihexanoyl-phosphatidyl-THIO (diC6-THIO) showed high anticancer activity with sub-µM EC50 values in vitro across various cancer cell lines. In mouse colorectal cancer models, diC6-THIO demonstrated strong anticancer effects alone and in combination with PD1/PD-L1 inhibitors. Administration of this compound resulted in the efficient formation of Telomere dysfunction Induced Foci (TIFs) in vitro, indicating an on-target, telomerase-mediated telomere-modifying mechanism of action for the molecule. Systemic treatment also activated CD4^+^ and CD8^+^ T cells while reducing regulatory T cells, indicating immune system enhancement. Notably, diC6-THIO exhibits an improved solubility profile while maintaining comparable anticancer properties, further supporting its potential as a promising therapeutic candidate. These findings highlight diC6-THIO as a promising telomere-targeting prodrug with dual effects on telomere modification and immune activation.

## 1. Introduction

Colorectal cancer (CRC) is the third leading cause of cancer-related deaths globally. The overall 5-year relative survival rate is 63%. In clinical settings, primary treatment options for colorectal cancer depend on the stage of the cancer and the patient’s individual circumstances. Even though the options are chemotherapy, targeted therapy, radiation therapy, immunotherapy, and surgery, treatment plans can change over time based on the patient’s response to therapy and disease progression [1,2]. Three FDA-approved immune checkpoint inhibitors (ICIs) targeting programmed cell death 1 (PD-1) and cytotoxic T lymphocyte antigen 4 (CTLA-4) (Pembrolizumab, Nivolumab, and Ipilimumab, respectively) are currently in clinical use for colorectal cancer [3]. Unfortunately, ICIs often provide a limited contribution to patient survival in clinical studies [4,5,6]. Therefore, it is crucial to find new therapeutic approaches and modalities to treat colorectal cancer that might also allow expanding the efficacy of the immunotherapies.

Targeted therapies, as opposed to non-specific chemotherapies, are designed to minimize adverse effects on normal tissues by targeting uniquely aberrant cancer markers. Combining targeted therapies and/or chemotherapies with prodrug strategies has shown some promises in cancer treatment [7]. Prodrugs are commonly characterized as pharmacologically inactive molecules. Nevertheless, their structural adaptability, achieved through modifications, facilitates conversion through enzymatic or chemical processes, leading to the release of bioactive agents—pharmacophores [8]. Fatty acids, cholesterol, glycerides, and phospholipids are some of the molecular entities that are used in the design of nanocarriers, since they have the capability of self-assembling into nanostructures. Small molecule lipidic prodrugs provide several advantages, including reported minimal toxicity, due to the endogenous fatty materials they contain, increased cellular uptake, and a better pharmacokinetic profile [9,10]. Due to these natural lipids being generally minimally or non-toxic, biocompatible, and biodegradable, lipid-based prodrugs emerge as attractive candidates for drug development [11,12,13,14].

Therefore, we proposed and now tested that utilization of the hydrophobic and hydrophilic properties of diacylglycerophospholipids may facilitate the improved solubility and cellular uptake of poorly water-soluble compounds and could allow the development of a new class of anticancer prodrugs.

Telomeres are found at the end of eukaryotic chromosomes and are composed of repetitive nucleotide sequences TTAGGG [15,16]. While telomeres shorten during each cell cycle in normal cells, they are elongated by the telomerase enzyme, which provides an unlimited proliferative capacity to most cancer cells [17]. The nucleoside analog 6-thio-2′deoxyguanosine (known as 6-thio-dG or THIO) is modified in its 5′-triphosphate form and is effectively recognized by telomerase and consequently incorporated into newly synthesized telomeric DNA products. This telomere modification by 6-thio-dG leads to TIFs (Telomere dysfunction Induced Foci) formation, also known as telomeric DNA damage signals. Since telomeres in humans are only about 1/6000th of the genome, any damage at telomeres is rare. Since approximately 85% of all tumors express telomerase activity [18], while the vast majority of normal untransformed cells are telomerase-negative, targeting telomerase-positive cancer cells through telomeric DNA modifications enhances anticancer treatment specificity toward cancer cells without significantly affecting normal cells [19,20].

**Figure 1:**
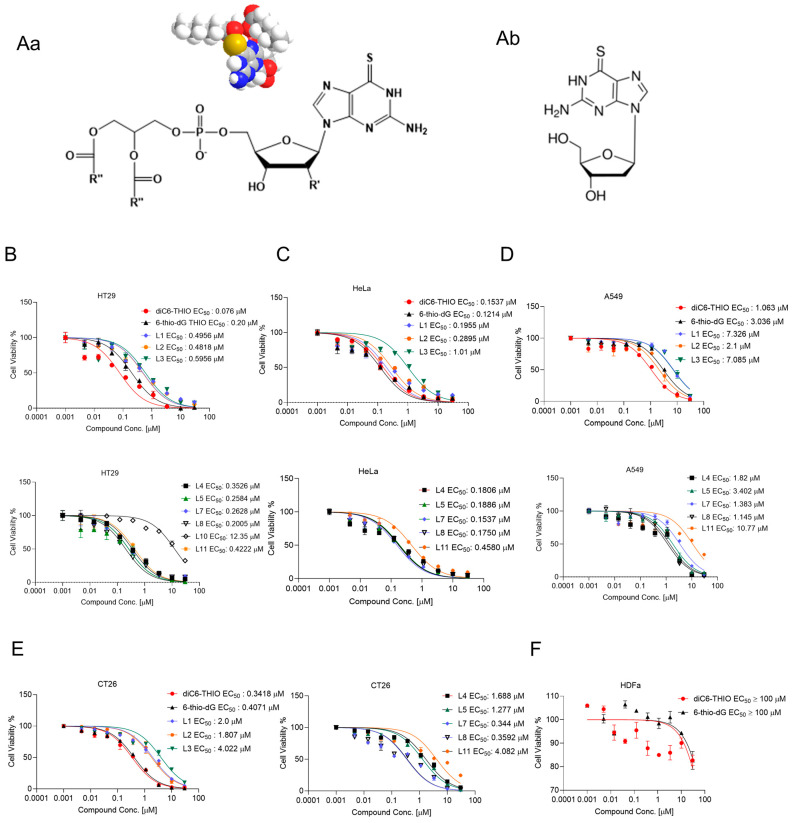
Biologic activity of phosphatidyl nucleoside conjugates in different human and murine cancer cell lines. General chemical structure of nucleoside phosphatidyl diglycerides, where R′ = H, and R″ = C3–C17 fatty acid residues; for diC6-THIOmolecule, R′ = H, R″ = C5 (**Aa**). Chemical structures of 6-thio-dG (**Ab**). Cell viability of human colorectal HT29 (**B**), human cervical HeLa (**C**), human NSCLC A549 (**D**), murine colorectal CT26 (**E**) cancer cell lines, and human dermal fibroblast HDFa cells (**F**) treated with the indicated concentrations of compounds for 4 days. Cell viability was measured using the MTT Assay. Samples were analyzed in triplicate, and EC_50_ values were calculated using GraphPad Prism.

In this work, we designed, prepared, and evaluated a series of new phospholipid-conjugated 6-thio-dG derivatives with variable lengths of fatty acid residues to assess their therapeutic effects both in cell cultures and in animal models. Dihexanoyl phosphatidyl 6-thio-dG conjugate (designated as diC6-THIO) showed higher cancer cell cytotoxicity in vitro compared to the other lipid conjugates. Therefore, we selected diC6-THIO for further in vivo evaluation as a potential candidate for advanced telomere-targeted therapies. The chemical structures of the studied phosphatidyl nucleoside conjugates are shown in Figure 1A. We conducted a series of in vitro and in vivo experiments to evaluate the therapeutic effect of diC6-THIO. These experiments confirmed the proposed mechanism of action, involving the induction of telomeric DNA damage in colorectal cancer models using the DiAna plugin methodology [21]. Following the effective dose-finding experiments in mice, we investigated the anticancer effects of diC6-THIO treatment in combination with anti-PD-L1 therapy using syngeneic immunocompetent CRC mouse models. Subsequently, we evaluated whether diC6-THIO could activate the host innate immune system and induce adaptive immune responses in the tumor microenvironment (TME) through flow cytometry analysis.

## 2. Materials and Methods

**Cell Lines:** Human colorectal (HT29), human cervical (HeLa), human non-small cell lung cancer (A549), parental human breast cancer (MDAMB-231 WT) and its brain metastatic derivative (BrM) and bone metastatic derivative (BoM-1833), human glioblastoma (U87), murine colorectal cancer (CT26), and a human dermal fibroblast (HDFa) cell strain were purchased from the ATCC. Murine colorectal carcinoma (MC38) cells were obtained from Nanjing Cobioer Biosciences Co., Ltd. (Nanjing, China). Lewis lung carcinoma (LLC) cells were obtained from the National Collection of Authenticated Cell Cultures. All cell lines were routinely evaluated using a mycoplasma contamination kit (R&D). HT29 and HeLa cells were cultured in Dulbecco’s Modified Eagle’s Medium (DMEM) (Serena, Pessin, Germany) supplemented with 10% fetal bovine serum (FBS) (Serena, Pessin, Germany); A549 and CT26 cell lines were cultured in Roswell Park Memorial Institute (RPMI) 1640 medium (Serena, Pessin, Germany) supplemented with 10% FBS (Serena, Pessin, Germany); MDAMB-231, BrM, and BoM-1833 cell lines were cultured in Media X with 10% FBS; HDFa cells were cultured in a 1:1 mixture of DMEM and Ham’s F-12 supplemented with 15% heat-inactivated FBS, 100 Unit/milliliter (U/mL) penicillin (Serena, Pessin, Germany), and 1% L-glutamine (Serena, Pessin, Germany); and MC38 and LLC cells were maintained in DMEM supplemented with 10% FBS. All cell lines were incubated under 5% CO_2_ at 37 °C.

**Drug Preparation:** Anti-mouse PD-L1 was purchased from BioCell (#BE0101; Lebanon, NH, USA), anti-mouse PD1 antibody was purchased from BioXCell (#BE0146), and 6-thio-dG and other phosphatidyl nucleoside conjugate compounds were supplied by MAIA Biotechnology (Chicago, IL, USA). For in vitro experiments, 6-thio-dG and other compounds were dissolved in 100% dimethyl sulfoxide (DMSO) (Merck, Darmstadt, Germany) to prepare stock solutions at 5 mM and 30 mM concentrations, respectively; it was stored at −20 °C. For in vivo experiments, 6-thio-dG (3 milligrams/kilogram; mg/kg) and diC6-THIO (3 mg/kg) were prepared in the mixture of 5% DMSO and 95% PBS for intraperitoneal (i.p.), or diC6-THIO (6 mg/kg) and sdiC6-THIO (6 mg/kg) were prepared in PBS for intravenous administration (i.v.). The anti-PD-L1 antibody (10F.9G2) was prepared at a concentration of 2.6 mg/mL (Bio X Cell, Lebanon, NH, USA) in PBS; 6-thio-dG, diC6-THIO, anti-PD-L1, and anti-PD-1 agents were stored at 4 °C. The anti-PD-1 antibody (clone: RMP1-14) was prepared at a concentration of 1 mg/mL in PBS and stored at 4 °C.

**Cell Viability Assay:** To assess the half-maximal effective concentration (EC_50_) of the compounds, both murine and human cancer cell lines were screened with 6-thio-dG and candidate compounds using a 3-fold dilution series across 9 different concentrations in 96-well plates. Cells were seeded 24 h before drug treatment and analyzed using the CellTiter Glo, MTT assay (1 mg/mL per cell) (Naturewill Biotechnology, Chengdu, Sichuan, China), or Cell Counting Kit-8 (CCK8) after a 4-day incubation period. The cell counts per well varied between 1000 and 5000 cells, depending on their doubling times. In the MTT, CellTiter Glo, and CCK8 assays, cell viability (%) was determined by normalizing the data to the control group, which was not treated with any compound. Data normalization in GraphPad Prism was conducted using the average values from six wells of the control group in a 96-well plate. This approach was consistently applied across the nine different conditions tested, with each of these nine doses represented by six replicates to ensure robust statistical analysis. Dose–response curves and EC_50_ values were generated using nonlinear regression analysis, specifically employing LogEC_50_ values for graphical representation of the dosage in logarithmic form. Samples were analyzed in triplicate, and the standard deviations were derived from a minimum of three independent experiments. Experiments were repeated a minimum of three times prior to determining the EC_50_ values to validate the findings.

**Droplet Digital TRAP (ddTRAP):** To measure the telomerase activity, cell pellets were prepared according to the ddTRAP protocol [22]. Briefly, cells were cultured to the desired density, counted (50,000 to 1 × 10^6^ cells), and pelleted. The pellets were either lysed immediately on ice or flash-frozen in liquid nitrogen and stored at −80 °C. Lysis was performed in 40 μL of NP-40 buffer for 30 min on ice. The lysate cell number equivalent was calculated by dividing the number of cells lysed by the volume of lysis buffer. One microliter of lysate was added to a 50 μL extension reaction containing 1× TRAP reaction buffer, 0.4 mg/mL BSA, 200 nM TS telomerase substrate, and dNTPs and incubated at 25 °C for 40 min, followed by inactivation at 95 °C for 5 min. The ddPCR reaction was prepared with 1× EvaGreen ddPCR Supermix v2.0, 50 nM TS, ACX primers, ≤50 cell equivalents of extension product, and dH_2_O 20 μL. Droplets were generated using a droplet generator and transferred to a 96-well PCR plate. PCR was performed with a ramp rate of 2.5 °C/s, including 40 cycles of 95 °C for 30 s, 54 °C for 30 s, and 72 °C for 30 s, followed by a final hold at 4 °C. Fluorescence was read using the 6-Fam channel on a droplet reader, analyzing an average of 17,000 droplets per 20 μL PCR. Telomerase activity was quantified as the number of extended TS molecules per microliter and normalized to cell equivalents. Background signal was assessed using a no-template control.

**Telomere Dysfunction Induced Foci (TIF) Assay:** HT29, HeLa, and CT26 cells were seeded into poly-lysine (Sigma, St. Louis, MO, USA)-coated wells. The cells were treated with 1 µM 6-thio-dG and 1 µM diC6-THIO for 96 h. The glass coverslips were washed with pre-extraction buffer (20 millimolar (mM) Tris-HCl, pH: 8, BioShop, Burlington, ON, Canada; 50 mM NaCl and 3 mM MgCl_2_, Sigma, Hamburg, Germany; 0.5% Triton X-100, BioShop, Canada; and 300 mM sucrose, Merck, Darmstadt, Germany) and fixed in a solution containing 4% formaldehyde (Thermo Fisher, Waltham, MA, USA). Then, the cells were permeabilized in a 0.5% solution of PBST (Triton X-100, BioShop Canada) and blocked with a 5% bovine serum albumin (BSA, Pessin, Germany) solution. The cells were incubated with the primary antibody (1:500, gammaH2AX, Cell Signaling, Danvers, MA, USA) and washed with 0.1% PBST. Subsequently, the cells were incubated with AlexaFluor 568 conjugated goat anti-mouse antibody (Invitrogen, Waltham, MA, USA) at a dilution of 1:1000 and fixed by 4% formaldehyde in PBS (Sigma, St. Louis, MO, USA) at room temperature. The slides were dehydrated with ethanol (Sigma, Hamburg, Germany) solutions of 70%, 90%, and 100%, and the slides were denaturated with hybridization buffer containing a peptide nucleic acid (PNA, Eurogentec, Seraing, Belgium) conjugated with fluorescein amidite (FAM). The samples were heated at 85 °C on a heat block for 4 min, washed with a washing solution (1M Tris HCl, pH: 7.5, 50% formamide; Sigma, Germany, 10% BSA), and mounted using Vectashield mounting medium with DAPI (Vector Laboratories, Newark, CA, USA).

TIF images were captured using a confocal microscope (Leica, sp8) with an oil immersion objective (63×, NA:1.4). For each region of interest, 50 × 50 µm^2^ areas were scanned at 512 × 512 pixel resolution, and Z-stacks with 0.3 µm steps were recorded, capturing the entire axial range of the cells to be analyzed. A total of 50 cells were analyzed for each condition. The raw data were further processed using ImageJ/FIJI (Version: 1.54b), and background subtraction and a gaussian filter with sigma = 1 pixel was applied. Quantitative analysis on these preprocessed images was then performed using the DiAna plugin [21] for testing object-based 3D colocalization between green and red channels. The threshold for maxima selection was set by the user and was readjusted by manually checking the segmentation results with the raw fluorescent image data, confirming that no artificial seeds were generated and at least 90% of the visible fluorescent foci are recognized as seed maxima. Once segmentation was complete for both channels, colocalization was determined by the detection of overlapping objects, and colocalizing object volumes were calculated for each pair of objects. The statistical significance of colocalization was calculated by comparing the experimental results with a random distribution, using the respective function of the DiAna plugin. The cumulative distribution of the mean distances between objects for experimental images and randomly shuffled images were compared, and the colocalizations were considered statistically significant if the experimental mean distance distribution curve was localized outside the 95% confidence interval of the randomized distance. Only statistically significant colocalization analyses are reported in the experimental results.

**Radiation Therapy Experiments:** HT29 cells (4000 cells/well) were seeded into 6-well plates (Nest Scientific, Woodbridge, NJ, USA) and treated with diC6-THIO (0.3 μM) and 6-thio-dG (0.3 μM) over a 96-h incubation period. After diC6-THIO and 6-thio-dG incubation, cells were irradiated with the Varian DHX linear accelerator (Varian Medical Systems, Palo Alto, CA, USA) operating at 6 MV X-ray energy, with doses of 2 Gray (Gy) and 4 Gy. Following the irradiation, the cells were cultured for an additional 24 h and subsequently counted using methylene blue staining.

**Spheroids Culture:** CT26 spheroids were generated by a free-floating spheroid culture system in PETG Erlenmeyer flasks in RPMI-1640 media. Single-cell suspensions were seeded into flasks as 5 × 10^5^ cells/mL and cultivated for 5 days. After checking and confirming the spheroid formation under a microscope, 1 μM and 3 μM diC6-THIO were added into the media and incubated for 96 h.

**Flow Cytometry Analysis for Spheroid Formation:** Flow cytometry was used to quantify the percentage of cell viability in CT26 spheroid cells treated with diC6-THIO compared to the control group. For each condition, 5 mL from the 25 mL cell/media solution was taken and centrifuged at 1000× *g* for 5 min. Supernatant was discarded, and 1 mL of Trypsin-EDTA (0.05%) was added to the cell pellet and incubated for 5 min at 37 °C. After the incubation, the trypsin solution was diluted 1:10 with base media. Single-cell suspension was verified under the microscope. Cells were centrifugated again at 1000× *g* for 5 min. The supernatant was removed, cells were washed twice with FACS buffer (PBS + 3% FBS), and centrifugated at 1000× *g* for 5 min each time. The supernatant was discarded and cells stained with eBioscience Fixable Viability Dye eFluor 506 (Ref: 65-0866-14) at a 1:50 dilution in FACS buffer and incubated for 30 min at 4 °C. After incubation, 1 mL of FACS buffer was added to dilute the dye and centrifuged at 1000× *g* for 5 min. The supernatant was removed, and cells were washed twice with FACS buffer and centrifugated at 1000× *g* for 5 min. Finally, the cell pellet was resuspended in 200 µL of FACS buffer and analyzed on the flow cytometer (Beckman CytoFLEX, Indianapolis, IN, USA). Sample concentration was adjusted as needed to maintain a flow rate of no more than 2500 events/minute. FSC (Forward Scatter) and SSC (Side Scatter) parameters are used to analyze and distinguish different cell populations based on their size and granularity.

**In vivo Experiments:** Male nude CD1 and BALB/c mice were purchased from Kobay Experimental Animal Laboratory. All mice were maintained under specific pathogen-free conditions. This study was approved by the Ethical Committee of the Kobay Experimental Animal Laboratory under protocol number 603. C57BL/6N female mice (6–9 weeks old) were purchased from Beijing Vital River Laboratory Animal Technology Co., Ltd., Beijing, China C57BL/6N animals were housed at HitGen Inc. (Chengdu, China) animal house facility.

**Xenograft Mouse Tumor Model:** To determine the optimal dose of diC6-THIO, 2 × 10^6^ human HT29 cells were injected into the right dorsal flanks of the six to eight-week-old male CD1 nude mice in 100 µL PBS. Tumor size measurements were conducted on a weekly basis, and treatment was started when the tumor volume reached 70–100 mm^3^. The mice were subjected to diC6-THIO treatment at dosages of 3 mg/kg (total of 6 doses on days 0, 2, 4, 6, 8, and 10, with day 0 designated as the day of treatment start), and 6 mg/kg (total of 4 doses on days 0, 2, 4, and 6, with day 0 designated as the day of treatment start). Tumor volumes were measured using a caliper by the length (a), width (b), and height (h) and calculated as tumor volume = a × b × h/2.

**Syngeneic Mouse Tumor Model:** A total of 2 × 10^6^ murine CT26 cells were injected into the left dorsal flanks of the six to eight-week-old male BALB/c mice in 100 µL PBS with 10% Matrigel (Corning, Corning, NY, USA). Tumor size measurements were conducted on a weekly basis, and treatment was started when the tumor volume reached 70–100 mm^3^ and designated here as day 0. In the CT26 model, tumor volumes were measured using a caliper by the length (a), width (b), and height (h) and calculated as tumor volume = a × b × h/2. When the tumor volumes reached 70–100 mm^3^, the animals were divided into 3 treatment groups. In the diC6-THIO treatment group, the mice were administered 3 mg/kg diC6-THIO twice a week over 2 weeks on days 0, 2, 7, and 9, with day 0 designated as the day of treatment start. In the anti-PD-L1 group, mice were administered weekly with 10 mg/kg anti-PD-L1 for two weeks on days 4 and 11. In the sequential therapy group, mice received diC6-THIO at a dosage of 3 mg/kg, twice weekly, over 2 weeks, coupled with a weekly administration of 10 mg/kg anti-PD-L1 (diC6-THIO on days 0, 2, 7, and 9 and anti-PD-L1 on days 4, 11). After the 2-week treatment period, mice from the control and diC6-THIO groups were euthanized, and their tumors were collected and analyzed via flow cytometry.

A total of 4 × 10^4^ murine MC38 or 2 × 10^6^ LLC cells were subcutaneously injected into the right dorsal flanks of the female C57BL/6N mice in 100 µL PBS (n = 8 per each group). Treatment started when the tumor volume reached 70–100 mm^3^ and was designated as day 0. In the treatment groups, the mice were administered with 6 mg/kg diC6-THIO (i.v.) or 6 mg/kg sdiC6-THIO (i.v.) on days 0, 1, 2, 7, 8, and 9 and/or 10 mg/kg anti-PD-1 (i.p.) on days 4 and 12. In the MC38 and LLC models, tumor length and width were measured using a caliper. Tumor volume = 0.5 × (length × width^2^).

**Flow Cytometry Analysis:** Freshly collected tumor tissues were first immersed in RPMI-1640 medium supplemented with 10% FBS. To prepare for flow cytometry, whole tumors were mechanically dissociated and subjected to enzymatic digestion to ensure optimal single-cell suspension. Specifically, the tumors were incubated in a digestion solution containing collagenase type II (100 U/mL, Nordmark, Hamburg, Germany) and DNase I (200 U/mL, Sigma-Aldrich, St. Louis, MO, USA) in RPMI-1640. The suspension was shaken in a 37 °C water bath for 1 h to achieve effective tissue breakdown. Following digestion, the dissociated cells were passed through a 40 μm sterile strainer (SPL Life Sciences, Pocheon, Republic of Korea) to obtain a single-cell suspension, rinsed with RPMI-1640 medium containing 10% FBS. The total number of cells per mg of tumor tissue was calculated for both the control and diC6-THIO groups, yielding 237,859 ± 9245 and 39,721 ± 4966 cells, respectively (AVG ± SEM). A consistent input of 5 × 10^6^ cells from the single-cell suspension was then taken for flow cytometry labeling. Cells were stained with specific antibodies targeting myeloid and T cell markers, including CD45-PerCP (BioLegend, San Diego, CA, USA), CD11b-APC/Cy7 (Sony Biotechnology, San Jose, CA, USA), F4/80-FITC (BioLegend, San Diego, CA, USA), Gr1-PE (BioLegend, San Diego, CA, USA), CD3-BV421 (BioLegend, San Diego, CA, USA), CD4-FITC (BioLegend, San Diego, CA, USA), CD8a-PE (BioLegend, San Diego, CA, USA), CD62L-APC (BioLegend, San Diego, CA, USA), and FoxP3-PE (BioLegend, San Diego, CA, USA). For intracellular FoxP3 staining, the True-Nuclear Transcription Factor Buffer Set (BioLegend, San Diego, CA, USA) was used according to the manufacturer’s instructions. After antibody incubation, cells were washed, and flow cytometry was conducted using a BD FACSCanto II. At least 10,000 cell events were recorded for the macrophage (CD45^+^CD11b^+^F4/80^+^) or lymphocyte (CD45^+^CD3^+^) population to ensure a robust analysis. Gating was set based on autofluorescence controls, and the data were analyzed using FlowJo 10 software. The gating strategy used to analyze the phenotypic distribution of immune cells infiltrating the tumor is shown in Supplementary Figure S2A,B.

**diC6-THIO Anti-PD-L1 and Sequential Therapy Effects on Liver and Kidney Functions:** To determine whether diC6-THIO, anti-PD-L1, and sequential therapy have a toxic effect on liver and kidney, we collected blood samples from treatment groups and controls, centrifuged at 2000× *g* for 10 min (Thermo Scientific, Megafuge 40R, Waltham, MA, USA). Serum samples were used for aspartate amino transferase (AST), alanine amino transferase (ALT), creatinine, and blood urea nitrogen (BUN) measurement (Beckman Coulter, AU680, Brea, CA, USA).

**Quantification and Statistical Analysis:** All the data analyses were performed with GraphPad Prism 8 and presented as mean ± SEM. Flowcytometry analysis was performed with FlowJo. *p*-value was determined by two-way ANOVA for xenograft and syngeneic mouse model tumor growth experiments. Two-way ANOVA multiple comparisons were used for TIF and global DNA damage assays. For flow cytometry analysis, the distribution of the groups was evaluated with the unpaired Student’s *t*-test. In the radiation experiment and liver, and kidney function tests, the *p* value was determined by unpaired *t*-test. *p* ≤ 0.05 was considered statistically significant.

## 3. Results

**Screening of Phosphatidyl Conjugated Compounds in Different Cancer Cell Lines**

To determine which fatty acid residues of glycerophospholipids provide the highest anticancer activity in vitro, we evaluated a series of phospholipid-conjugated compounds using HT29, HeLa, A549, CT26, MC38, LLC, HDFa, MDAMB-231 WT, BrM, BoM-1833, and U87 cells. The chemical structures of diC6-THIO and 6-thio-dG are shown in Figure 1Aa and Figure 1Ab, respectively. We found the corresponding EC_50_ values to be 0.2 µM, 0.49 µM, 0.48 µM, 0.59 µM, 0.35 µM, 0.25 µM, 0.076 µM, 0.26 µM, 0.20 µM, 12.35 µM, and 0.42 µM for 6-thio-dG and L1, L2, L3, L4, L5, L6 (diC6-THIO), L7, L8, L10, and L11, respectively, in HT29 cells (Figure 1B–E). We found that other phospholipid-conjugated molecules also show efficacy like diC6-THIO in these cancer cell lines (summarized in Table 1) and thus may potentially be evaluated as a second generation of telomerase-mediated telomere-targeted candidate molecules in future preclinical studies. Consequently, we continued our preclinical studies with the most active molecule, diC6-THIO, and compared the compound’s in vitro activity against 6-thio-dG. We found that the tested telomerase-positive cancer cells were sensitive to both diC6-THIO (EC_50_ values: 0.076–3.527 µM) and 6-thio-dG (EC_50_ values: 0.12–3.036 µM) (Table 1). However, diC6-THIO showed higher cytotoxicity in HT29 and A549 cancer cells as compared to 6-thio-dG (Figure 1B–D). Furthermore, we evaluated the effects of these compounds on the metastatic cell lines BrM and BoM-1833 and compared the results to their parental MDAMB-231 cell line. Our findings indicate that both metastatic cancer cell lines BrM and BoM-1833 are more sensitive to 6-thio-dG compared to their respective parental cell line. Additionally, the BoM-1833 metastatic cell line shows enhanced sensitivity to diC6-THIO relative to the parental MDA-MB-231 cells (Supplementary Figure S7C). At the same time, the EC_50_ values for both diC6-THIO and 6-thio-dG were above 100 µM in telomerase-negative normal human dermal fibroblast cells (HDFa) (Figure 1F), demonstrating the specificity of both compounds to telomerase-positive cancer cells vs. their normal untransformed counterparts.

**Metastatic Cell Sensitivity to diC6-THIO Correlates with Increased Telomerase Activity**

diC6-THIO is incorporated into de novo synthesized telomeres by telomerase. To assess this mechanism and correlate the EC_50_ values with telomerase activity in metastatic cell lines, we performed the ddTRAP assay. Our results showed that the parental MDAMB-231 cell line exhibited lower telomerase activity compared to its metastatic derivatives (Supplementary Figure S7C). These findings suggest that the enhanced sensitivity of the metastatic derivative of MDAMB-231, BoM-1833, to diC6-THIO is associated with elevated telomerase activity.

**diC6-THIO Treatment Induces Telomere Dysfunction Induced Foci (TIF) Formation**

**Figure 2:**
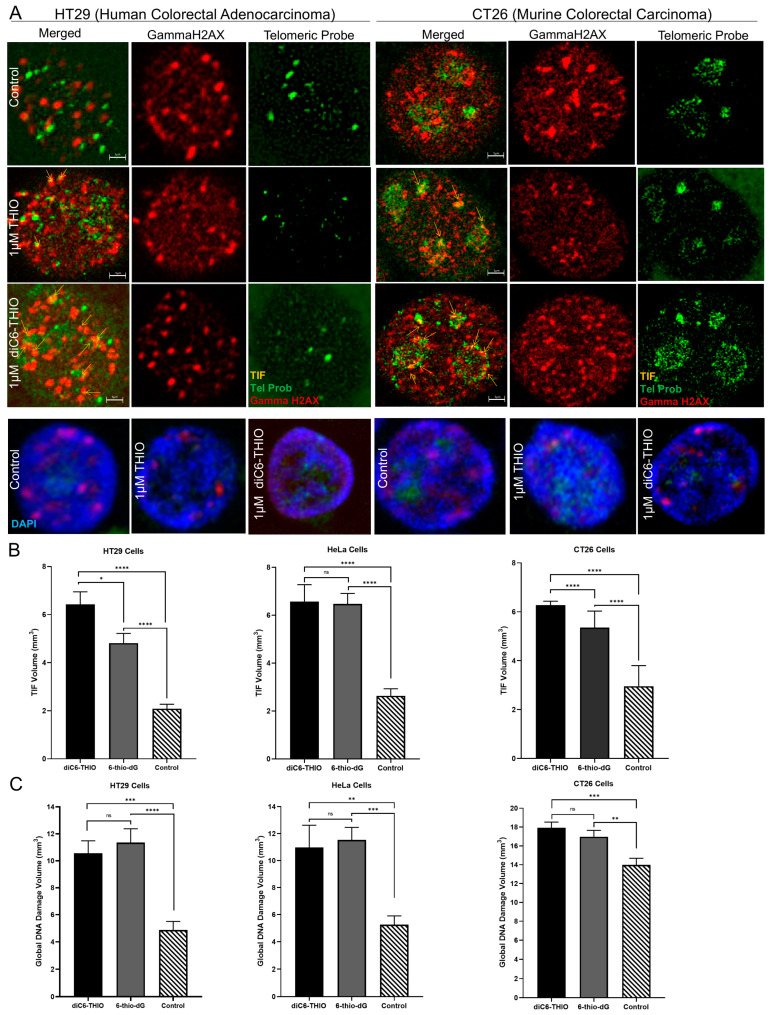
diC6-THIOinduces more TIFs compared to 6-thio-dG. Representative 2D images of TIF and DNA damage foci for diC6-THIO and 6-thio-dG in HT29 and CT26 cells with 1 μM treatment for 4 days. Green: Telomeric probe, red: gammaH2AX, yellow: TIFs, and blue: DAPI (**A**). Merged images with arrows show the representative pictures of TIFs (**A**); the quantitative measurements of TIF volumes (**B**); and global DNA damage (**C**) of HT29, HeLa, and CT26 cells treated with diC6-THIO (1 μM) and 6-thio-dG (1 μM) for 4 days. Data are shown as means ± SEM from two to three independent experiments. *p*-value was determined by two-way ANOVA followed by a post hoc test (Tukey’s). All TIF and global DNA damage volumes were scored by DiAna plugin (n ≈ 50 for HT29, HeLa, and CT26 cells. *p*-values for TIF between control vs. 6-thio-dG (**** *p* < 0.0001) or control vs. diC6-THIO (**** *p* < 0.0001) or 6-thio-dG vs. diC6-THIO (* *p* = 0.0147) in HT29; control vs. 6-thio-dG (**** *p* < 0.0001) or control vs. diC6-THIO (**** *p* < 0.0001) or 6-thio-dG vs. diC6-THIO (*p* = 0.9966) in HeLa; and control vs. 6-thio-dG (**** *p* < 0.0001) or control vs. diC6-THIO (**** *p* < 0.0001) or 6-thio-dG vs. diC6-THIO (**** *p* < 0.0001) in CT26. ns, not significant. *p*-values for global DNA damage between control vs. 6-thio-dG (**** *p* < 0.0001) or control vs. diC6-THIO (*** *p* = 0.0001) or 6-thio-dG vs. diC6-THIO (*p* = 0.8267) in HT29; control vs. 6-thio-dG (*** *p* = 0.0004) or control vs. diC6-THIO (** *p* = 0.0014) or 6-thio-dG vs. diC6-THIO (*p* = 0.9314) in HeLa; and control vs. 6-thio-dG (** *p* = 0.0077) or control vs. diC6-THIO (*** *p* = 0.0003) or 6-thio-dG vs. diC6-THIO (*p* = 0.5879) in CT26. ns, not significant.

To evaluate whether the diC6-THIO compound induces telomere damage in cancer cells, we treated HT29, HeLa, and CT26 cells with diC6-THIO and 6-thio-dG at 1 µM concentration for 96 h. Colocalization of the signals derived from genomics, and telomeric DNA damage foci was determined by the detection of overlapping objects (Supplementary Figure S1A (left)). These data showed that the segmentation tools exclude some spots that are not relevant to the TIF foci and capture colocalization areas (Supplementary Figure S1A (right)). In addition to colocalization measurements, the DiAna technique [21] provided detectable objects in the respective images (Supplementary Figure S1B). The DiAna plugin algorithm generates a graphical representation of the cumulative distribution of the minimum center-to-center distances between objects in two different images (images for gammaH2AX and telomeres), as illustrated in Supplementary Figure S1C. Within the graphs, the blue curves correspond to the distribution observed in the experimental images. The green curves represent the confidence interval centered around the mean. Meanwhile, the red curve shows the mean distribution of the distances between objects from the experimental images and images obtained through the shuffle procedure. Significantly, the experimental blue curve lies beyond the 95% confidence interval (green) of the distance analysis following randomization. Hence, this colocalization analysis is considered statistically significant. Both 6-thio-dG and diC6-THIO induced a considerable number of telomere damage (TIFs) in all three cell lines evaluated. At the same time, treatment with diC6-THIO generated a significantly higher number of TIFs compared to 6-thio-dG in both HT29 and CT26 cancer cells (Figure 2A,B). The increase in the number of TIFs may be attributed to the presence of a dihaxanoyl phosphatidyl group in the diC6-THIO structure, providing for a better cellular uptake of the molecule in HT29 and CT26 colorectal cancer cells and affecting intracellular pharmacophore’s metabolism and distribution. However, no significant difference in TIFs induction was observed between diC6-THIO and 6-thio-dG in HeLa ovarian cancer cells, where the compounds’ EC_50_ values were also comparable to each other (Figure 2B).

In addition, both diC6-THIO and 6-thio-dG induced global genomic DNA damage (as assessed by induction of gammaH2AX DNA damage foci) in HT29, HeLa, and CT26 cells, where there was no significant difference between diC6-THIO and 6-thio-dG treatment groups (Figure 2C). Our findings indicate that diC6-THIO exhibits higher TIFs induction compared to that of 6-thio-dG in colorectal cancer cells and thereby higher degree of telomere alteration, resulting in an increased activity in vitro.

**diC6-THIO-induced Dissociation of CT26 Spheroids**

Sphere culture, which facilitates the entrapment and enrichment of cancer stem cells (CSCs), is widely regarded as a highly effective method for isolating CSCs from cancer cell lines and solid tumors [23,24]. Additionally, spheroid cultures provide an optimal platform for the routine assessment of toxicity and drug efficacy, facilitating the determination of safe exposure doses in well-established cellular models [25,26]. We aim to investigate the impact of diC6-THIO on spheroid formation. Our results demonstrated that CT26 cell-derived spheroids were sensitive to diC6-THIO treatment, leading to their dissociation (Supplementary Figure S7D). This dissociation suggests that diC6-THIO may disrupt intercellular adhesion and potentially enhance the efficacy of other therapeutic modalities.

**diC6-THIO Reduces Tumor Growth in HT29-Derived Xenograft and CT26 Syngeneic Mouse Models**

**Figure 3:**
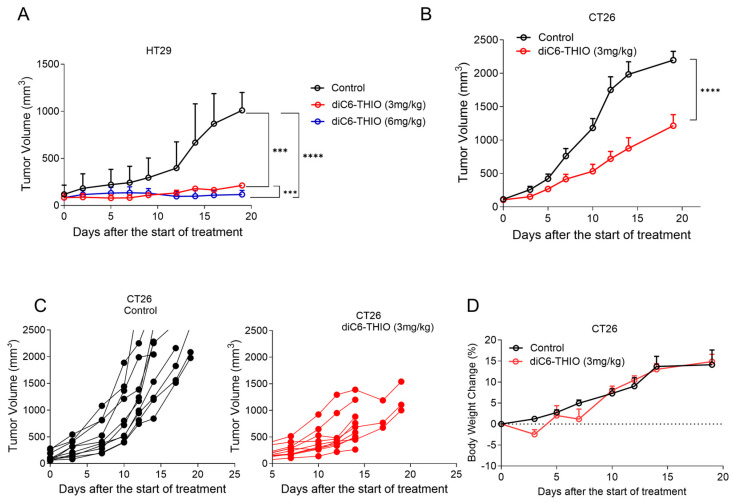
diC6-THIO reduces tumor growth in xenograft and syngeneic mouse models. Xenograft model with HT29 cells. The mice were subjected to 3 mg/kg diC6-THIO treatment (total of 6 doses on days 0, 2, 4, 6, 8, and 10, with day 0 designated as the day of treatment start) and 6 mg/kg diC6-THIO treatment (total of 4 doses on days 0, 2, 4, and 6, with day 0 designated as the day of treatment start). Tumor volumes were scored by GraphPad Prism (n = 2 per each group for nude CD1 mice, 2 × 10^6^ HT29 cells were injected). *** *p* = 0.0003 (control vs. 3 mg/kg diC6-THIO), **** *p* < 0.0001 (control vs. 6 mg/kg diC6-THIO), and *** *p* = 0.0008 (3 mg/kg diC6-THIO vs. 6 mg/kg) in two-way ANOVA, (control; untreated) (**A**). The BALB/c mice tumor volume measurements. 2 × 10^6^ murine CT26 cells were injected. BALB/c mice bearing CT26 tumors were treated with diC6-THIO (3 mg/kg, days 0, 2, 7, and 9, with day 0 designated as the day of treatment start). Data are shown as means ± SEM from two independent experiments. *p*-value was determined by two-way ANOVA by using GraphPad Prism. (n = 10 per each group, **** *p* < 0.0001 control vs. diC6-THIO in two-way ANOVA, control; untreated) (**B**). Individual tumor growth of control and diC6-THIO treatment groups (**C**). Graph shows body weight changes of mice in percentage following diC6-THIO treatment. The weights were measured every 2 days (**D**).

We have previously shown that 6-thio-dG controls tumor growth with daily or every other day treatment for over 10 days as a monotherapy in xenograft immunodeficient mice cancer models [27]. Here, we evaluated the treatment of the diC6-THIO compound in CD1 nude male mice at 3 mg/kg and 6 mg/kg doses, where the compound injections were done intraperitoneally (i.p.) every other day for 6–10 days. Although the 6 mg/kg dose resulted in animal weight loss after four administrations, 3 mg/kg dose was well tolerated. We thus extended the treatment period with 3 mg/kg doses of diC6-THIO for up to six injections. Significant tumor reduction was observed in both the 3 mg/kg and 6 mg/kg dose groups when compared to the control group (Figure 3A). These results led us to identify the 3 mg/kg dose as the optimal therapeutic dose level for the following in vivo studies. Then, we evaluated diC6-THIO at the 3 mg/kg dose level in a CT26-derived immunocompetent syngeneic mouse model of colorectal cancer. CT26 cells were inoculated into BALB/c mice, and 14 days after inoculation (when the tumor volume reached ~70–100 mm^3^), diC6-THIO (3 mg/kg) was administered twice a week for 2 weeks (on days 0, 2, 7, and 9, with day 0 corresponding to the day of the treatment initiation). Tumor volumes were monitored and measured for 2 weeks. We observed that tumor growth was significantly reduced in the CT26-derived immunocompetent model by the treatment with diC6-THIO, without any significant animal weight loss (Figure 3B–D).

**diC6-THIO Treatment Reduces Tumor Growth Compared to Control and Anti-PD-L1 Therapy**

**Figure 4:**
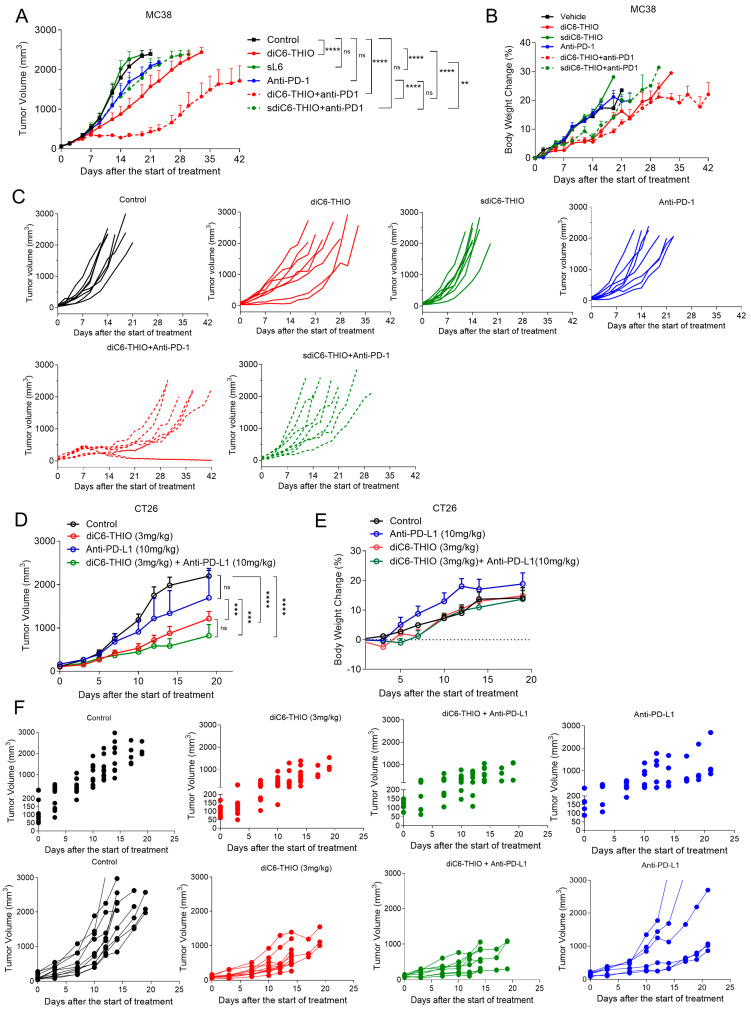
Therapeutic efficacy of diC6-THIO when sequentially combined with anti-PD-1 and anti-PD-L1 in MC38 and CT26 colon cancer models. Data are shown as means ± SEM. *p*-value was determined by two-way ANOVA by using GraphPad Prism. In MC38 mouse model treatment groups, the mice were administered with 6 mg/kg diC6-THIO (i.v.) or 6 mg/kg sdiC6-THIO (i.v.) on days 0, 1, 2, 7, 8, and 9 and/or 10 mg/kg anti-PD-1 (i.p.) on days 4 and 12 (n = 8 per group). There were statistically significant differences between control vs. diC6-THIO (**** *p* < 0.0001), control vs. diC6-THIO + anti-PD-1 (**** *p* < 0.0001), diC6-THIO + anti-PD-1 vs. anti-PD-1 (**** *p* < 0.0001), control vs. diC6-THIO + anti-PD-1 (**** *p* < 0.0001), anti-PD-1 vs. DIC6-THIO + anti-PD-1 (**** *p* < 0.0001), diC6-THIO vs. diC6-THIO + anti-PD-1 (**** *p* < 0.0001), sdiC6-THIO vs. sdiC6-THIO + anti-PD-1 (** *p* = 0.0013), and diC6-THIO vs. sdiC6-THIO (**** *p* < 0.0001). No significant differences (ns) were found between control vs. sdiC6-THIO (*p* = 0.9301), control vs. anti-PD-1 (*p* = 0.1357), control vs. sdiC6-THIO + anti-PD-1 (*p* = 0.0756), and anti-PD-1 vs. sdiC6-THIO + anti-PD-1 (*p* = 0.9995). For statistical calculations, the final measurements from the euthanized mice are included until the completion of each group, which is determined by the endpoint reached when all mice in that group die. When comparing two groups, the statistical calculations consider the endpoint of the earlier group as reference (**A**). The body weight changes in percentage from MC38 control, diC6-THIO, sdiC6-THIO, diC6-THIO+ anti-PD-1, sdiC6-THIO + anti-PD-1, and anti-PD-1 groups (**B**). Individual MC38 tumor growth curves from control and treatment groups (**C**). In the CT26 mouse model, the mice were administered with 3 mg/kg diC6-THIO (i.p.) on days 0, 2, 7, 9 and/or 10 mg/kg anti-PD-L1 (i.p.) on days 4, 11. there was statistically significant difference between control vs. diC6-THIO (**** *p* < 0.0001), control vs. diC6-THIO + anti-PD-L1 (**** *p* < 0.0001), diC6-THIO vs. anti-PD-L1 (*** *p* = 0.0009), and diC6-THIO + anti-PD-L1 vs. anti-PD-L1 (*** *p* = 0.0003). No significant differences were found between diC6-THIO vs. diC6-THIO + anti-PD-L1 (*p* = 0.3222) and control vs. anti-PD-L1 (*p* = 0.4222). For statistical purposes only, the final measurements from the euthanized mice were included up to the completion of each group, which occurred on day 19 (**D**). The body weight changes of mice in percentage from CT26 control, diC6-THIO, diC6-THIO + anti-PD-L1, and anti-PD-L1 groups (**E**). Individual CT26 tumor growth curves from control and treatment groups (**F**).

We evaluated the therapeutic efficacy of the active compound diC6-THIO and its metabolically more stable and inactive control form, designated as sdiC6-THIO, in a MC38-derived syngeneic mouse model. While diC6-THIO contains natural phosphodiester (P-O) linkage, where it connects the fatty acid residues of the lipid groups to 6-thio-dG, sdiC6-THIO contains enzymatically more stable non-natural modified phosphorothioate (P-S) linkage (Supplementary Figure S5A). The specific chemical nature of the P-S linkage makes this compound more stable in cells; thus, the nucleoside pharmacophore 6-thio-dG is not readily released from the sdiC6-THIO prodrug in cells. Cell viability assays of MC38 and LLC cells confirmed the differences in biological activity of these compounds in vitro. Two independent in vitro experiments conducted in MC38, and LLC cells showed 14–120-fold difference in activity between diC6-THIO and sdiC6-THIO (Supplementary Figure S5B,C). When we compared the compounds’ efficacies in vivo, we found significant tumor growth delay with diC6-THIO alone. However, its metabolically less active form sdiC6-THIO did not show any therapeutic effect compared to PBS-treated controls. In addition, we compared diC6-THIO and sdiC6-THIO-treated animals to their ICI combinational counterparts—groups with sequential treatments of diC6-THIO and anti-PD-1 or sdiC6-THIO and anti-PD-1 agents. Sequential treatment with diC6-THIO and anti-PD-1 compounds showed significant differences when compared to the control, diC6-THIO alone, and anti-PD-1 alone, without noticeable animal weight loss. Sequential treatment with sdiC6-THIO and anti-PD-1 did not show significant differences when compared to control or anti-PD-1 agents alone. Sequential treatment with sdiC6-THIO and anti-PD-1 had significant differences when compared to sdiC6-THIO alone (Figure 4A–C).

Our next step was to investigate the therapeutic efficacy of the diC6-THIO compound, the anti-PD-L1 antibody and the sequential therapy of diC6-THIO and anti-PD-L1 in a CT26-derived syngeneic mouse model. Previous studies have highlighted distinct immune responses between the MC38 and CT26 colorectal cancer models, underscoring the importance of selecting appropriate checkpoint inhibitors based on tumor characteristics. Zhong et al. demonstrated that CT26 tumors respond robustly to CTLA-4 checkpoint inhibitors but show limited response to PD-1 inhibitors [28]. Carretta et al. conducted a comparative analysis and showed that CT26 cells exhibit higher PD-L1 gene expression than MC38 cells, suggesting potential differences in their responsiveness to PD-L1-targeted therapies [29]. Their analysis of the tumor microenvironment (TME) composition via flow cytometry further revealed substantial variations in immune and stromal cell infiltration between the two models. Specifically, MC38 tumors are significantly more infiltrated by immune cells than CT26 tumors, contributing to their distinct immunogenic profiles. Given these findings, we decided to use anti-PD-L1 therapy with CT26 cells rather than anti-PD-1, considering the higher PD-L1 expression and unique TME characteristics of CT26 tumors.

We measured tumor volumes until tumor sizes reached approximately 2500 mm^3^. While there was no significant tumor growth inhibition with anti-PD-L1 as a monotherapy, diC6-THIO alone or with sequential treatment of diC6-THIO and anti-PD-L1 showed a significant difference compared to control with no noticeable animal weight loss (Figure 4D–F). There were statistically significant differences between control vs. diC6-THIO (**** *p* < 0.0001), control vs. diC6-THIO + anti-PD-L1 (**** *p* < 0.0001), diC6-THIO vs. anti-PD-L1(*** *p* = 0.0009), and diC6-THIO +anti-PD-L1 vs. anti-PD-L1 (*** *p* = 0.0003). No statistically significant difference was observed between diC6-THIO vs. diC6-THIO + anti-PD-L1 and control vs. anti-PD-L1. While no statistically significant difference was observed between the diC6-THIO and sequential therapy group, the trend suggests that the sequential therapy involving diC6-THIO and anti-PD-L1 holds the potential to decrease tumor volume.

In addition, aspartate transaminase (AST) and alanine transaminase (ALT) plasma levels were evaluated for liver function (Supplementary Figure S4A), and creatinine and urea nitrogen (BUN) plasma levels were evaluated for renal function (Supplementary Figure S4B). Even though the treatment groups did not alter creatinine and BUN compared to the control, the ALT and AST levels were decreased in the treatment groups compared to the control.

We also evaluated the therapeutic efficacy of diC6-THIO and sdiC6-THIO in a different cancer model. We selected a LLC cell-based murine lung cancer model and confirmed the similar therapeutic effects of diC6-THIO in this alternative model. Once again, we lost the therapeutic efficacy when we used the inactive sdiC6-THIO form of the molecule. The sequential administration of diC6-THIO and anti-PD-1 resulted in significant differences in the LLC model when compared to diC6-THIO or anti-PD-1 groups alone. The sequential treatment of sdiC6-THIO and anti-PD-1 did not show significant differences when compared to sdiC6-THIO or anti-PD-1 alone (Supplementary Figure S5D–F). These results indicate that a hydrolytically less stable P-O intermodular linkage allows for the release of 6-thio-dG from the prodrug and provides for therapeutic activity. Additionally, diC6-THIO combination with anti-PD-1 agent provides additional therapeutic efficacy not only in colorectal but also in lung cancer models.

**diC6-THIO Treatment Enhances Activated CD4^+^ and CD8^+^ T Cells and Decreases T Regulatory Cells in the Tumor Microenvironment**

**Figure 5:**
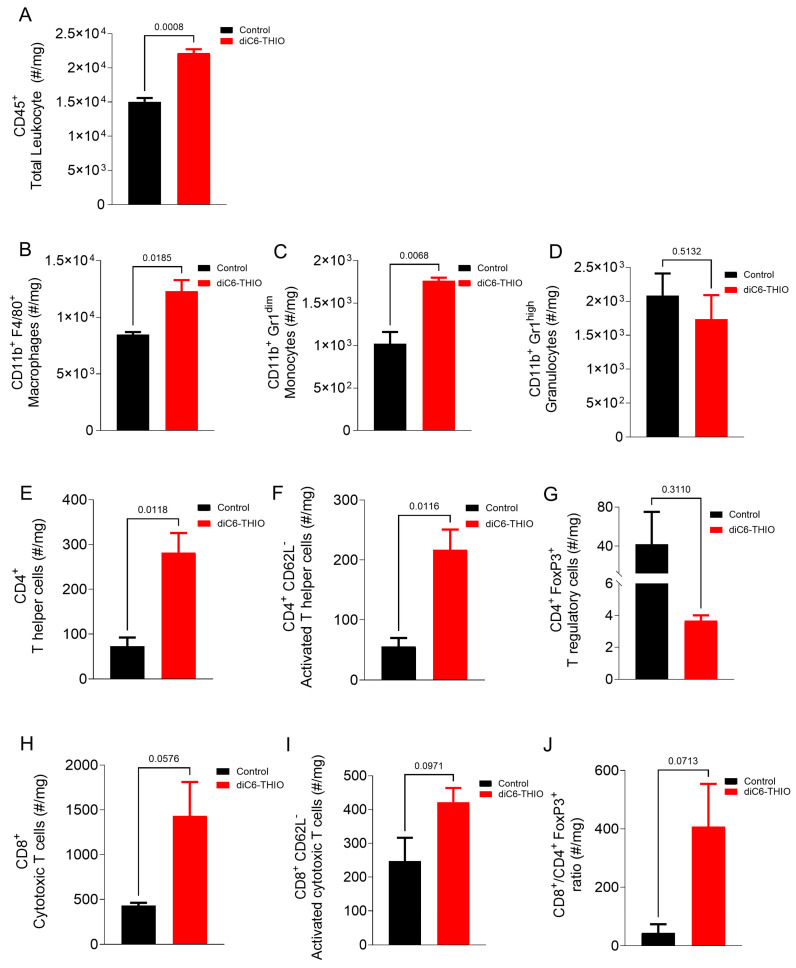
Immunophenotyping of CT26 bearing mice after diC6-THIO treatment. Total leukocyte (**A**) subpopulations. Myeloid subpopulations (**B**–**D**), lymphocyte subpopulations (**E**–**I**), and cytotoxic T cells/T regulatory cells ratio (**J**) in tumor tissue (number of cells in tumor tissue(#)/mg). Data are shown as means ± SEM. *p*-values were determined by unpaired Student’s *t*-test by using GraphPad Prism. Despite the lack of statistical significance among the groups for CD8^+^, CD8^+^ CD62L^−^, CD8^+^ CD4^+^ FoxP3^+^, and CD4^+^ FoxP3^+^ panels (*p* > 0.05), the trend for T helper and cytotoxic T cells were indicated that diC6-THIO has potential to induce activated T cell infiltration (**E**,**F**,**H**,**I**). Opposite, in the treatment group, T regulatory cell numbers decreased (**G**). Following diC6-THIO treatment cytotoxic T cells: T regulatory cells ratio increased (**J**).

We conducted flow cytometry to characterize immune cell populations within the tumor microenvironment. The initial analysis of immune cell percentages revealed no significant differences in the proportion of infiltrating immune cells in the diC6-THIO-treated group compared to the control group (Table 2, Supplementary Figure S3A–F). However, percentage-based comparisons alone may not fully capture changes in immune cell infiltration, particularly given the differences in tumor sizes between the groups. While the percentages provide valuable insights into the relative distribution of immune cell subsets, they do not account for absolute changes in cell infiltration per milligram of tumor tissue. To address this limitation, we quantified immune cell infiltration as the number of cells per milligram of tumor tissue (Figure 5). This analysis revealed that diC6-THIO treatment led to an increase in the total leukocyte (CD45^+^ cells) counts within the tumor tissue (Figure 5A). Among myeloid populations, macrophages and monocytes showed increased infiltration (Figure 5B,C), whereas granulocyte numbers remained unchanged (Figure 5D). Similarly, lymphoid populations exhibited enhanced infiltration of T helper (CD4^+^) and cytotoxic T cells (CD8^+^), accompanied by an increase in number of activated lymphocytes in both subsets (Figure 5E,F,H,I). In contrast, T regulatory (Treg) cell counts were reduced (Figure 5G). Importantly, the CD8^+^ T cell to Treg (#/mg CD8^+^/Treg) of (CD4^+^ FoxP3^+^) ratio increased following diC6-THIO treatment (Figure 5J). Although these findings suggest enhanced immune activation, the reductions in Tregs, increases in activated CD8^+^ T cells, and the elevated CD8^+^/Treg ratio did not reach statistical significance.

## 4. Discussion

Various drug delivery systems have been developed to enhance cellular uptake to achieve higher drug accumulation to improve cancer treatment outcomes. Chemotherapies, due to insufficient target cancer cell specificity, cause toxicity in healthy tissues and, thus, limit their therapeutic efficiency. To overcome these obstacles, the prodrug approaches have evolved to achieve more tumor-specific drug delivery and reduce toxic side effects [30].

In previous studies, a phospholipid–doxorubicin conjugate showed high drug loading content and facilitated cell internalization [31]. Another study also showed the successful conjugation of fatty acid, squalenoic acid (SQ) to gemcitabine, paclitaxel, doxorubicin, and adenosine [32,33,34,35]. Sauraj et al. [36] designed an effective delivery approach for 5-fluorouracil based on xylan–stearic acid conjugates and showed higher cellular apoptosis in vitro in colorectal cancer cells, thus showing the advantages of small lipid prodrugs-based drug delivery systems. Deoxycytidine analog gemcitabine has a short circulation half-life due to metabolic instability and poor diffusion into tumor cells due to drug resistance development and its hydrophilic moieties. Wu et al. [37] developed a prodrug with PUFAylation technology that contains gemcitabine and hydrophobic linoleic acid via amide linkage. This study showed that gemcitabine was protected from rapid deactivation by cytidine deaminase and increased the in vitro cytotoxicity in L3.6pl and BXPC-3 pancreatic cancer cells compared to non-conjugated gemcitabine.

Here, we designed and evaluated a series of new phosphatidyl-lipid-modified nucleoside conjugated bimodular molecules. We evaluated various cancer cell lines, including metastatic derivatives, to compare their responses to the dihexanoyl phosphatidyl derivative of 6-thio-dG, referred to as diC6-THIO. We demonstrated that metastatic cells derived from MDAMB-231 exhibit increased telomerase activity, as assessed by the highly quantitative ddTRAP assay [22], and show increased sensitivity to diC6-THIO treatment. The ddTRAP assay demonstrates that elevated telomerase activity increases cellular sensitivity to both 6-thio-dG and diC6-THIO. This observation is further supported by the EC_50_ data, which show that BrM and BoM cells, characterized by higher telomerase activity, exhibit greater sensitivity to both compounds. Overall, there was little difference in sensitivity between diC6-THIO and 6-thio-dG across these cell types. However, these results indicate that other tumor types are very sensitive to both compounds. Since diC6-THIO is more soluble compared to 6-thio-dG, this is a significant advantage to progress diC6-THIO in additional pre-clinical and in the future into clinical trials.

To further investigate this relationship, a correlation analysis was performed to assess the relationship between telomerase activity and sensitivity to drug treatment for both 6-thio-dG and diC6-THIO. The correlation analysis for 6-thio-dG revealed a Pearson correlation coefficient of r = −0.7852, indicating a moderately strong negative correlation between telomerase activity and EC50 values. This suggests that as telomerase activity increases, EC50 values tend to decrease, implying that cells with higher telomerase activity may be more sensitive to 6-thio-dG, as reflected by lower EC50 values. However, while the *p*-value for this analysis showed a trend, it did not reach statistical significance.

For diC6-THIO, the correlation analysis yielded a Pearson correlation coefficient of r = −0.9474, demonstrating a strong negative correlation between telomerase activity and EC50 values. This indicates a more pronounced relationship for diC6-THIO compared to 6-thio-dG, with EC_50_ values decreasing more significantly as telomerase activity increases. The proximity of the r value for diC6-THIO to −1 further suggests that this compound may be more effective in enhancing drug sensitivity by targeting telomerase activity (Supplementary Figure S7E). While this correlation approached lack of statistical significance, further studies with larger sample sizes are needed to confirm and expand these findings. These results reinforce our observation that tumors with elevated telomerase activity may show enhanced sensitivity to diC6-THIO.

After evaluating the EC_50_ values across different cell lines, we selected colorectal cancer cell lines for in vivo studies, as diC6-THIO demonstrated enhanced anticancer activity in these cells and exhibited improved solubility in aqueous biological media. Phospholipid conjugated prodrugs typically have a therapeutic agent, a phosphate group, and a glycerol residue esterified with fatty acids as their main components. The diC6-THIO molecule also consists of 6-thio-dG as a telomerase-dependent telomere-targeting pharmacophore, a phosphate group linker and hexanoyl diglyceride as the main compound structural components. The telomere-targeting activity of diC6-THIO is associated with the induction of enhanced telomere damage (TIFs), which is one of the most accurate pharmacodynamic markers of the compound mechanism of action and on-target activity indicator. Since human telomeres are only about 1/6000th of the entire genome, damage to telomeres only rarely occurs. We employed a novel ImageJ-based technique, called DiAna, capable of conducting spatial analysis in three dimensions to calculate the spatial separation between colocalized object volumes for every selected pair of objects (Supplementary Figure S1B). Colocalization analysis is performed using a pixel-based method [38,39], an object-based method [40,41,42], or a hybrid approach that integrates both techniques [43]. In the pixel-based approach, colocalization analysis is affected by the inherent noise of fluorescent images and lacks the ability to provide spatial information concerning the relationship between objects. Meanwhile, object-based approaches have limitations as the threshold is globally applied to the whole image. Within the DiAna tool, we implemented 3D segmentation analysis and demonstrated precise and robust object extraction despite variations in object size and intensity across the images. The segmentation facilitates the precise calculation of the spatial volume occupied by an object in 3D space, along with the determination of the object’s geometric centroid or center of mass. Notably, this method effectively circumvents the inherent biases associated with 2D image analysis. The advantages of this method include its reduced susceptibility to actual pixel intensity values, robustness in handling background noise and its freedom from dependence on arbitrarily defined intensity threshold values. Using this analysis method, we found that diC6-THIO induces more TIFs formation compared to the unconjugated parent pharmacophore 6-thio-dG. This indicates a potential role for the lipid conjugated group in targeting telomeres, more efficiently in telomerase positive cells.

Moreover, diC6-THIO was evaluated in combination with radiation therapy in HT29 colorectal cancer cells in vitro. Pretreatment with diC6-THIO for 24 h followed by radiation therapy resulted in increased toxicity compared to treatment with diC6-THIO or radiation therapy alone at both 2 Gy and 4 Gy doses of ionizing radiation (Supplementary Figure S6A,B).

In cancer, intrinsic sensing deficits restrict T cell activation in the tumor microenvironment. Hence, innovative approaches and therapeutic agents are needed for combination therapies targeting patients both innate and adaptive immune systems to overcome this limitation. Currently, PD-1/PD-L1 blockade benefits are limited and require additional therapy or novel compounds and their combinations to overcome anti-PD-L1 agents’ resistance. Our in vivo immunophenotyping data revealed that diC6-THIO treatment significantly enhanced tumor infiltration of activated T cells while reducing the presence of Treg cells in tumor tissues. This was accompanied by an increase in the cytotoxic T cell/Treg cell ratio, where cytotoxic T cells serve as an important indicator of anticancer efficacy. These findings align with the observed reduction in tumor volumes following diC6-THIO treatment, suggesting an enhanced immune-mediated antitumor response. Importantly, the combined analysis of percentage-based and absolute cell counts provided a more comprehensive understanding of these changes. While the percentage-based data showed minimal differences in immune cell distribution (Table 2), absolute quantification demonstrated significant increases in immune cell infiltration per milligram of tumor tissue in the diC6-THIO-treated group. This dual analysis highlights the importance of integrating proportional and absolute metrics to accurately evaluate immune responses in the tumor microenvironment. The observed increase in immune cell counts per milligram of tumor tissue suggests that diC6-THIO promotes immune cell recruitment and activation, despite the stability in proportional distributions, supporting its potential as an immunomodulatory agent in cancer therapy. Collectively, these results indicate that diC6-THIO holds promise for clinical application, either as a standalone treatment or in combination with immune checkpoint inhibitors, particularly for colorectal cancer patients.

Taken together, our results demonstrate that the telomerase-mediated telomere-targeted anti-cancer effects for the bimodular prodrug 6-thio-dG-lipid-conjugate (diC6-THIO) is more pronounced than those for the unconjugated nucleoside pharmacophore in colorectal cancer models. In conclusion, we have introduced a new telomerase-mediated telomere-targeted glycerophospholipid-based prodrug that holds promise for a high anticancer therapeutic efficacy in vivo. Anticancer activity of this class of telomere-modifying prodrugs will require additional studies in other tumor xenograft and syngeneic mouse models.

## 5. Conclusions

We demonstrated potent and specific anticancer activity of a novel telomerase activity-dependent telomere-targeting small molecule, diC6-THIO. This compound is effective in telomerase-expressing cancer cells but not in normal telomerase-negative fibroblasts. Within this class of conjugates, diC6-THIO emerges as one of the most potent molecules, inducing significant telomeric DNA damage and exhibiting enhanced anticancer activity. Importantly, diC6-THIO has high solubility in aqueous media compared to the parental 6-thio-dG compound, which is currently in phase II clinical trials for lung cancer. Our findings in colorectal cancer models provide proof-of-principle results demonstrating the efficacy of diC6-THIO both in vitro and in vivo. Furthermore, we show that diC6-THIO treatment increases the proportion of infiltrated immune cells within the tumor microenvironment, suggesting its potential to enhance therapeutic responses to immunotherapies for colorectal cancer treatment.

In conclusion, diC6-THIO represents a phospholipid-conjugated derivative of 6-thio-dG with comparable anticancer activity and enhanced solubility. These features underscore its potential as an alternative therapeutic agent for cancer treatment.

## Figures and Tables

**Table 1:** Summarizing the EC_50_ values of all compounds evaluated in different cell lines following a 4-day treatment.

EC_50_ (µM)								
Compounds	HT29	HeLa	A549	CT26	MC38	LLC	HDFa	U87
**6-thio-dG**	0.2	0.1214	3.036	0.4071	1.507	0.172	>100	0.8985
**L1**	0.4956	0.1955	7.326	2	-	-	-	-
**L2**	0.4818	0.2895	2.1	1.807	-	-	-	-
**L3**	0.5956	1.01	7.085	4.022	-	-	-	-
**L4**	0.3526	0.186	1.82	1.688	-	-	-	-
**L5**	0.2584	0.1886	3.402	1.277	-	-	-	-
**L6 (diC6-THIO)**	0.076	0.1537	1.063	0.3418	3.527	0.3418	>100	0.7878
**sdiC6-THIO**	-	-	-	-	>50	23.94	-	-
**L7**	0.2628	0.1537	1.383	0.344	-	-	-	-
**L8**	0.2	0.175	1.145	0.3592	-	-	-	-
**L10**	12.35	-	-	43.07	-	-	-	-
**L11**	0.4222	0.458	10.77	4.02	-	-	-	-

**Table 2:** Percentage distribution of immune cell subpopulations in tumor tissues: Control vs. diC6-THIO treatment.

	Control	diC6-THIO
**CD45^+^ in total cells**	6.3 ± 0.2	5.6 ± 0.1
**CD11b^+^F4/80^+^ in CD45^+^**	69.4 ± 1.9	70.9 ± 2.4
**CD11b^+^Gr1^dim^ in CD45^+^**	8.4 ± 1.3	10.2 ± 0.3
**CD11b^+^Gr1^high^ in CD45^+^**	16.9 ± 2	10.3 ± 2.6
**CD4^+^ in CD45^+^**	3.4 ± 0.7	6 ± 1.6
**CD4^+^CD62L^−^ in CD3^+^**	77.9 ± 7.8	77 ± 0.2
**CD4^+^FoxP3^+^ in CD3^+^**	6.2 ± 2.7	0.5 ± 0.1
**CD8^+^ in CD45^+^**	21.5 ± 3.1	26.5 ± 1.9
**CD8^+^CD62L^−^ in CD3^+^**	58.5 ± 16.8	31.9 ± 4.8

Population values are presented as percentages, expressed as the mean ± standard error of the mean (SEM).

## Data Availability

The datasets generated during and/or analyzed during the current study are available from the corresponding author on reasonable request.

## Supplementary Materials

The following supporting information can be downloaded at https://www.mdpi.com/article/10.3390/biom14121616/s1: Figure S1: Telomere Dysfunction-Induced Foci (TIF) Formation Following diC6-THIO and 6-thio-dG Treatment. (A) Colocalization of signals from genomic and telomeric DNA damage foci in HT29, HeLa, and CT26 cells treated with diC6-THIO and 6-thio-dG (1 µM, 96 h). Left panel: Representative images showing colocalization. Right panel: Segmentation tools exclude irrelevant spots and capture colocalization areas. (B) Detection of overlapping objects using the DiAna technique, providing quantitative data on telomere-associated DNA damage foci. (C) Cumulative distribution of minimum center-to-center distances between gammaH2AX and telomere objects. Experimental data (blue curve) lie outside the 95% confidence interval (green curve) of shuffled images (red curve), indicating significant telomere damage; Figure S2: Gating strategy for the analysis of immune cell phenotypes infiltrating the tumor microenvironment. Panel A depicts the gating approach for the myeloid immune cell panel, while Panel B outlines the gating strategy for the lymphoid immune cell panel; Figure S3: Flow Cytometry Analysis of Tumor-Infiltrating Immune Cells in Control and diC6-THIO-Treated Groups; Figure S4: Evaluation of liver and renal function. (A) Plasma levels of aspartate transaminase (AST) and alanine transaminase (ALT) for liver function. (B) Plasma levels of creatinine and blood urea nitrogen (BUN) for renal function; Figure S5: Therapeutic Efficacy of diC6-THIO and sdiC6-THIO in Tumor Growth and Response to Anti-PD-L1 Therapy. (A) Chemical structure of diC6-THIO and sdiC6-THIO, highlighting the differences in phosphodiester (P-O) and phosphorothioate (P-S) linkages. (B,C) In vitro cell viability assays comparing the biological activity of diC6-THIO and sdiC6-THIO in MC38 and LLC cells, showing a 14–120-fold difference in efficacy. (D–F) In vivo tumor growth in MC38 and LLC murine models, demonstrating the therapeutic efficacy of diC6-THIO compared to sdiC6-THIO and the enhanced response to sequential diC6-THIO and anti-PD-1 therapy. The combination of sdiC6-THIO and anti-PD-1 did not show significant therapeutic effects; Figure S6: Enhanced toxicity of diC6-THIO in combination with radiation therapy in HT29 colorectal cancer cells. Increased cytotoxicity observed in HT29 cells following 24-h pretreatment with diC6-THIO, followed by ionizing radiation (2 Gy and 4 Gy), in contrast to the effects of diC6-THIO or radiation therapy alone; Figure S7: EC50 Values of Metastatic and Parental Cell Lines Treated with diC6-THIO and 6-thio-dG, Spheroid Formation in CT26 Cells, and Correlation between Telomerase Activity and Drug Sensitivity. (A) EC50 values of MDA-MB-231 parental cell line and its metastatic derivatives, BrM and BoM-1833, treated with diC6-THIO and 6-thio-dG. (B) EC50 values of U87 cells treated with diC6-THIO and 6-thio-dG. (C) ddTRAP analysis of telomerase extension products per cell in MDA-MB-231 parental (WT) and metastatic derivative cell lines. The figure demonstrates the correlation between telomerase activity and drug sensitivity. Increased telomerase activity correlated with higher drug sensitivity, as reflected by EC50 values. (D) Sensitivity of CT26 cell-derived spheroids to diC6-THIO treatment and its impact on spheroid dissociation. (E) Pearson correlation analysis between telomerase activity and EC50 values for 6-thio-dG and diC6-THIO treatment, demonstrating a stronger negative correlation for diC6-THIO.
