# Supplementary figures and images for: A Phosphatidyl Conjugated Telomerase-Dependent Telomere-Targeting Nucleoside Demonstrates Colorectal Cancer Direct Killing and Immune Signaling
Source: Biomolecules. 2024 Dec 18;14(12):1616. doi: 10.3390/biom14121616 (PMC11674679; doi:10.3390/biom14121616)

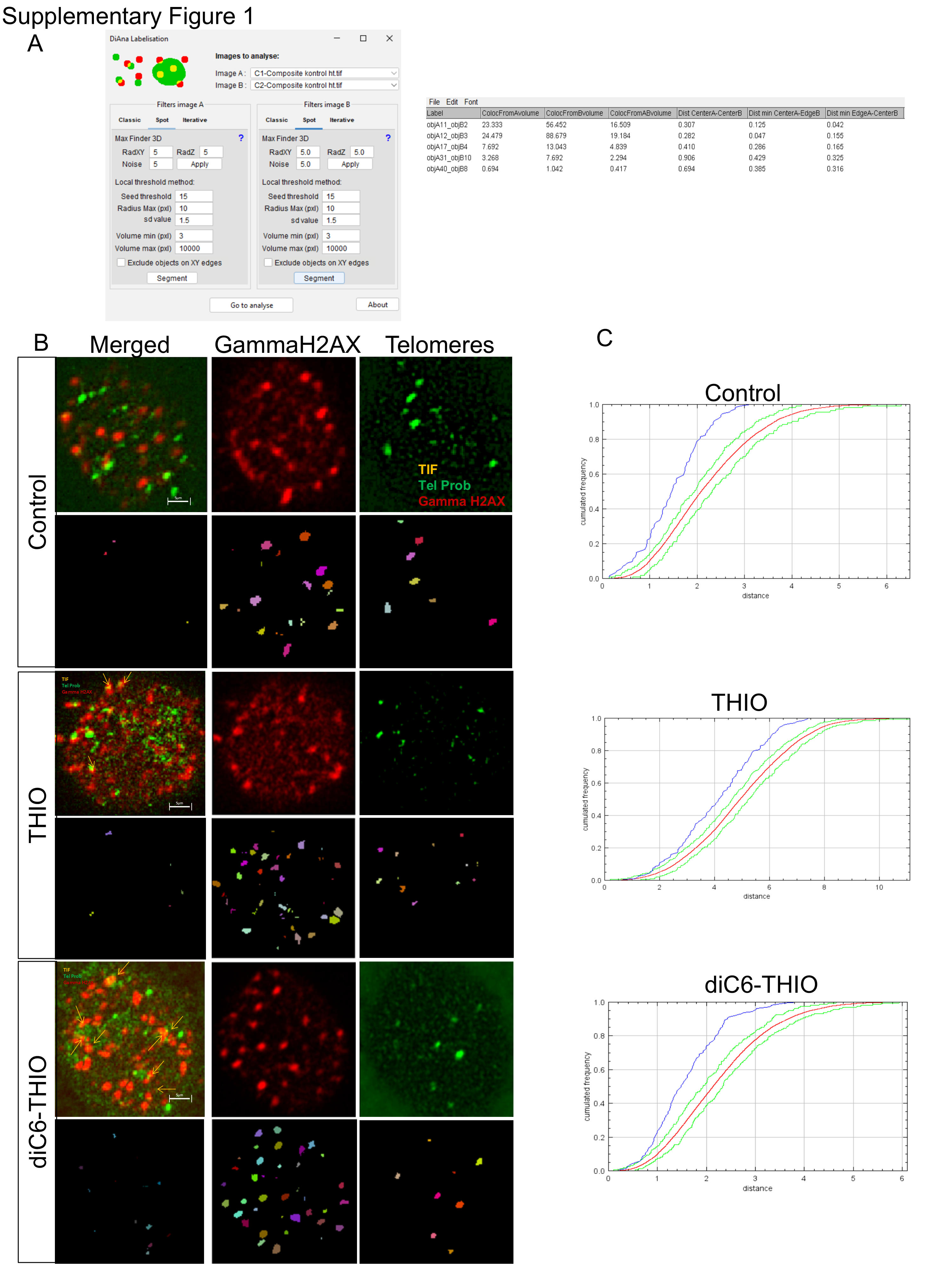

Supplement: Supplementary file 1 [file biomolecules-14-01616-s001.zip › Supplementary Figure S1.PNG]

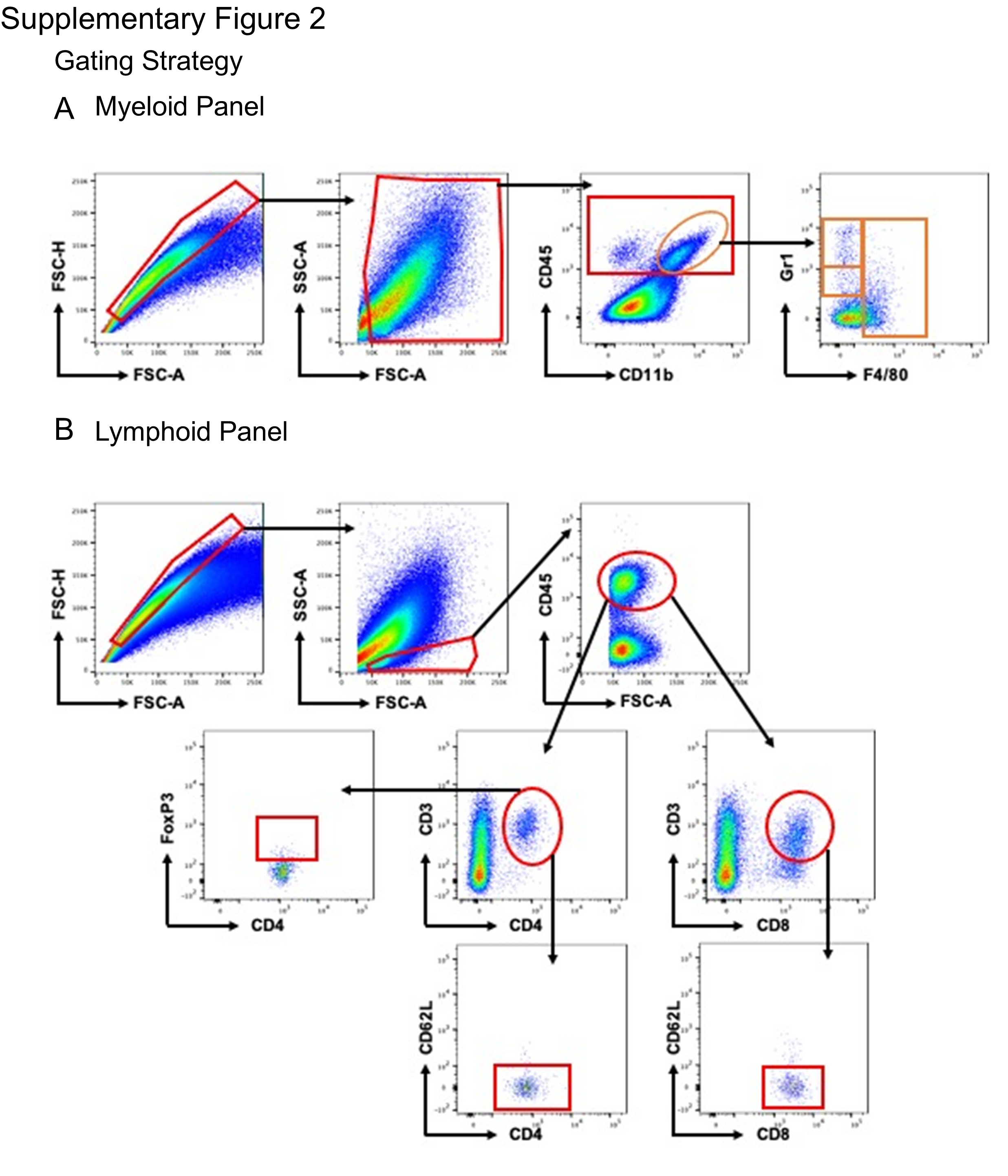

Supplement: Supplementary file 1 [file biomolecules-14-01616-s001.zip › Supplementary Figure S2.PNG]

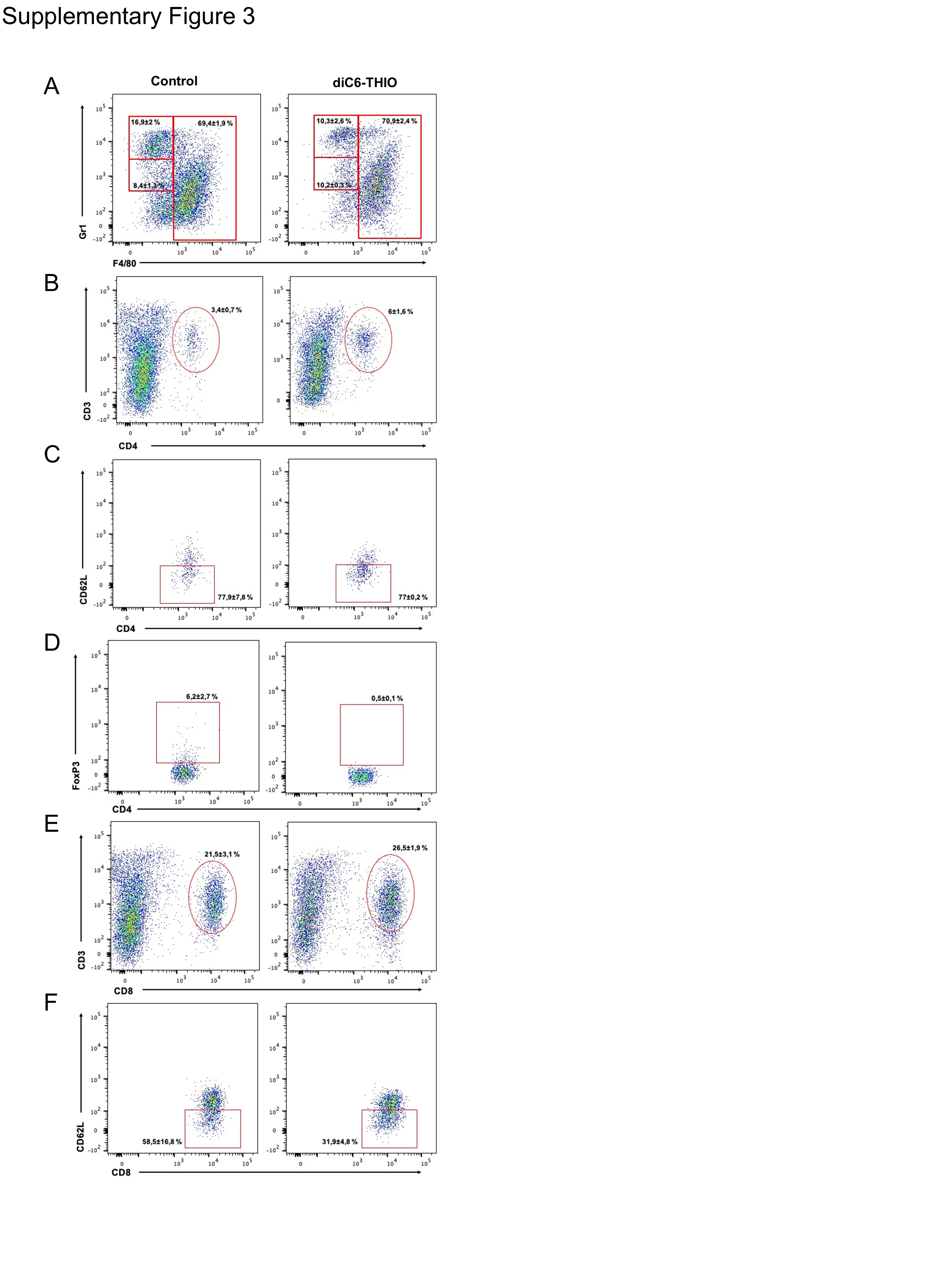

Supplement: Supplementary file 1 [file biomolecules-14-01616-s001.zip › Supplementary Figure S3.PNG]

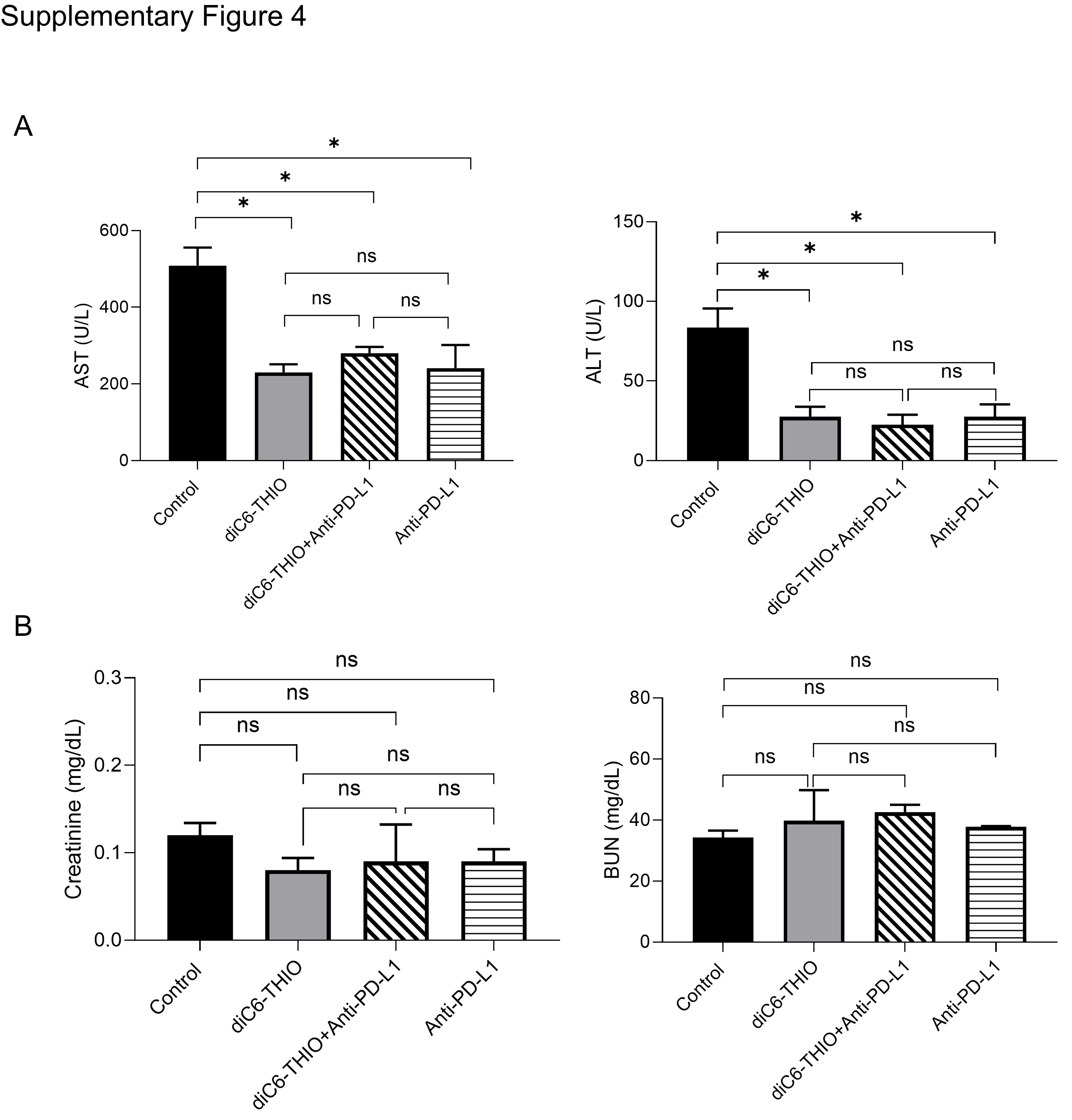

Supplement: Supplementary file 1 [file biomolecules-14-01616-s001.zip › Supplementary Figure S4.PNG]

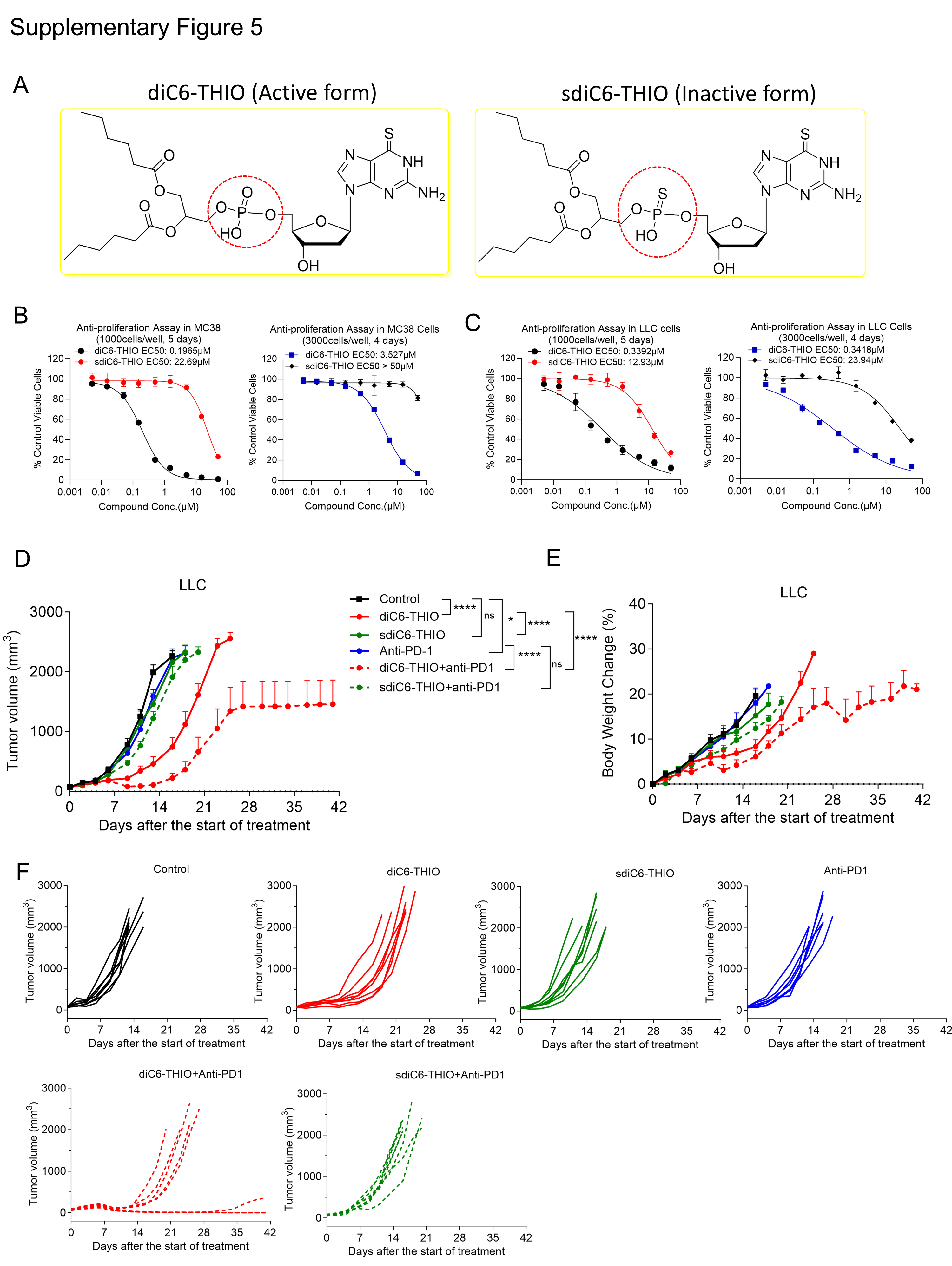

Supplement: Supplementary file 1 [file biomolecules-14-01616-s001.zip › Supplementary Figure S5.png]

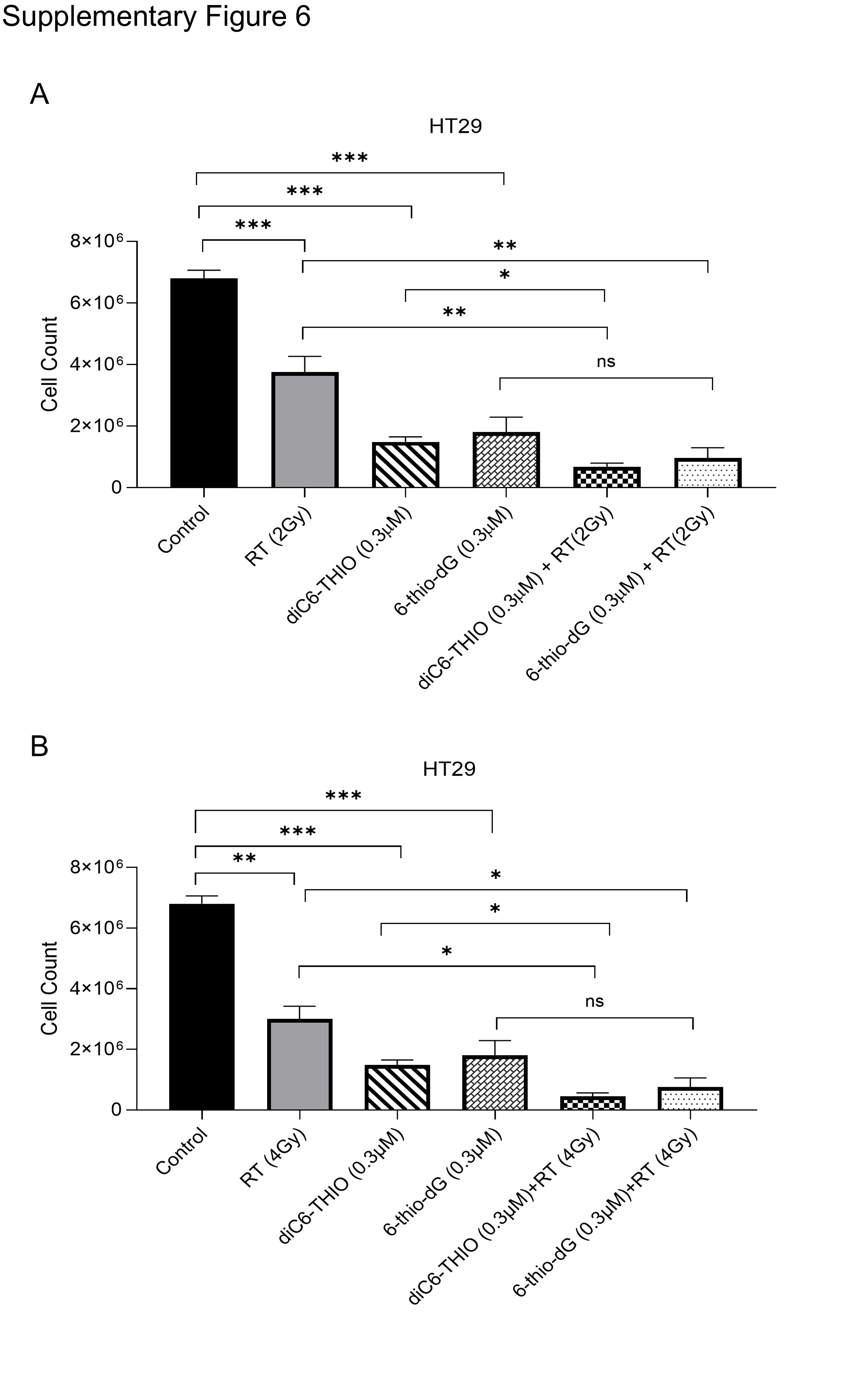

Supplement: Supplementary file 1 [file biomolecules-14-01616-s001.zip › Supplementary Figure S6.PNG]

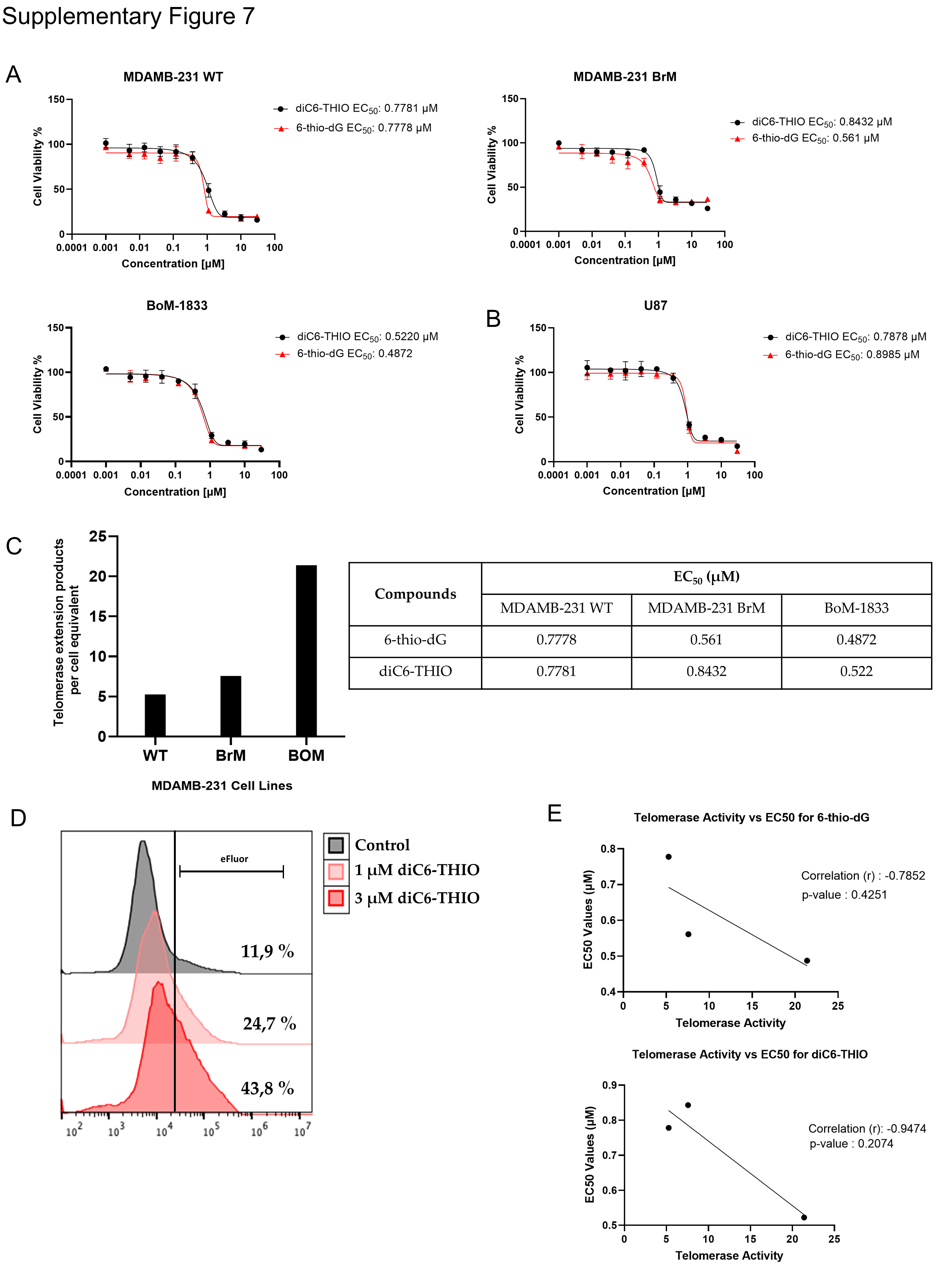

Supplement: Supplementary file 1 [file biomolecules-14-01616-s001.zip › Supplementary Figure S7.png]
